# Spatio-temporal analysis of leprosy risks in a municipality in the state of Mato Grosso-Brazilian Amazon: results from the leprosy post-exposure prophylaxis program in Brazil

**DOI:** 10.1186/s40249-022-00943-7

**Published:** 2022-02-22

**Authors:** Lúbia Maieles Gomes Machado, Emerson Soares dos Santos, Arielle Cavaliero, Peter Steinmann, Eliane Ignotti

**Affiliations:** 1 Institute of Public Heath, Post-Graduation Program in Public Health, Federal University of Mato Grosso, Cuiabá, Mato Grosso Brazil; 2 Department of Geography, Post-Graduation Program of Geography, Federal University of Mato Grosso, Cuiabá, Mato Grosso Brazil; 3 Novartis Foundation, Basel, Switzerland; 4 Swiss Tropical and Public Health Institute, Allschwil, Switzerland; 5 University of Basel, Basel, Switzerland; 6 School of Medicine, Post-Graduation Program in Health Sciences, Federal University of Mato Grosso, Cuiabá, Mato Grosso Brazil; 7 School of Health Sciences, Post-Graduation Program in Environment Sciences, State University of Mato Grosso, Cáceres, Mato Grosso Brazil

**Keywords:** Leprosy, Epidemiological profile, Contact tracing, Spatial analysis, Poverty, Surveillance

## Abstract

**Background:**

Leprosy post-exposure prophylaxis (LPEP) with single dose rifampicin (SDR) can be integrated into different leprosy control program set-ups once contact tracing has been established. We analyzed the spatio-temporal changes in the distribution of index cases (IC) and co-prevalent cases among contacts of leprosy patients (CP) over the course of the LPEP program in one of the four study areas in Brazil, namely the municipality of Alta Floresta, state of Mato Grosso, in the Brazilian Amazon basin.

**Methods:**

Leprosy cases were mapped, and socioeconomic indicators were evaluated to explain the leprosy distribution of all leprosy cases diagnosed in the period 2016–2018. Data were obtained on new leprosy cases [Notifiable diseases information system (Sinan)], contacts traced by the LPEP program, and socioeconomic variables [Brazilian Institute of Geography and Statistics (IBGE)]. Kernel, SCAN, factor analysis and spatial regression were applied to analyze changes.

**Results:**

Overall, the new case detection rate (NCDR) was 20/10 000 inhabitants or 304 new cases, of which 55 were CP cases among the 2076 examined contacts. Changes over time were observed in the geographic distribution of cases. The highest concentration of cases was observed in the northeast of the study area, including one significant cluster (Relative risk = 2.24; population 27 427, *P*-value < 0.001) in an area characterized by different indicators associated with poverty as identified through spatial regression (Coefficient 3.34, *P*-value = 0.01).

**Conclusions:**

The disease distribution was partly explained by poverty indicators. LPEP influences the spatial dynamic of the disease and results highlighted the relevance of systematic contact surveillance for leprosy elimination.

**Graphical Abstract:**

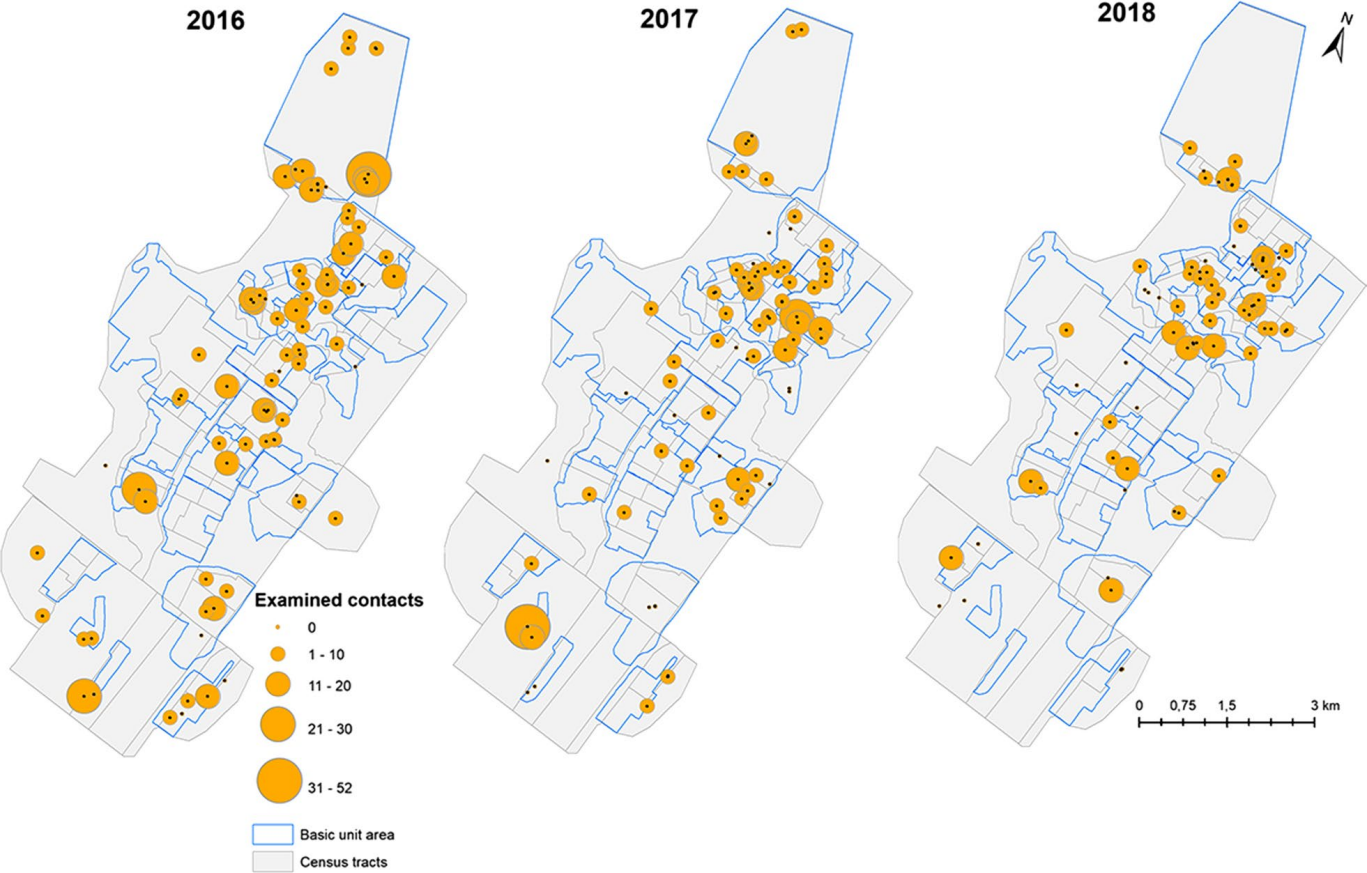

**Supplementary Information:**

The online version contains supplementary material available at 10.1186/s40249-022-00943-7.

## Background

The global distribution of leprosy is focused on South America, Africa, and South Asia, where mainly socioeconomically disadvantaged and marginalized populations are affected [1]. Brazil is endemic for this neglected tropical disease (NTD) and presents a heterogeneous spatial distribution. The Ministry of Health (MoH) manages specific programs to eliminate/control NTDs including leprosy. Mato Grosso state was historically classified as hyperendemic and remains the Brazilian state with the highest new case detection rate (NCDR). In 2019, Mato Grosso reported 129.3 cases per 100 000 inhabitants, almost 10 times more than the national NCDR, which stands at 13.2 per 100 000 inhabitants [2, 3]. The national leprosy program defines the protocol for diagnosis, treatment, and surveillance of leprosy. However, municipalities are responsible for the implementation of activities through their primary health care system, in coordination with specialized or reference centers. Technical support and drug supply are organized by the state, with coordination and support from federal levels. All cases of leprosy are treated within the Unified Health System (*Sistema Único de Saúde*—SUS) with national coverage [4].

Leprosy contact tracing is the basis of active surveillance and an important tool for the early diagnosis of new cases. It also contributes to transmission interruption through the elimination of possible sources of infection [5, 6]. Tracing and screening contacts is therefore part of any active case finding strategy, and it is currently recommended to include spatial analysis tools in endemic areas to support control programs [7, 8].

Chemoprophylaxis has been shown to be an effective tool for reducing the risk of developing leprosy among individuals exposed to *M. leprae* including close contacts of newly diagnosed patients [9]. Among other efforts to innovate and implement new strategies for the control and elimination of leprosy, in 2014 the Leprosy Post-Exposure Prophylaxis (LPEP) program was created, with implementation in seven countries: Brazil, India, Indonesia, Myanmar, Nepal, Sri Lanka and Tanzania. The World Health Organization (WHO) followed all steps of this initiative as observer. LPEP explored the feasibility and impact of tracing and screening contacts of new patients, as well as offering a single dose of rifampicin to eligible contacts. The program was implemented between 2016 and 2018 and covered a total of 180 000 contacts in different health systems [10–12]. The global strategic document was co-created to offer an aligned foundation across all LPEP countries on the scope and implementation of the LPEP program [10, 13].

In Brazil, the LPEP program documented the second highest NCDR among screened contacts across all LPEP countries (141 cases/10 000 contacts). The integration of post-exposure prophylaxis with single dose rifampicin (SDR-PEP) was shown to be safe, feasible, and acceptable for the routine of different leprosy control programs [12]. In Brazil, the LPEP protocol was implemented under the name “*PEP-Hans Brasil*” and also included the administration of the Bacille Calmette-Guérin (BCG) vaccine to eligible contacts, following the current protocol for leprosy surveillance in Brazil [4]. The MoH and Novartis Foundation (NF) supported the costs in Brazil. The State University of Mato Grosso (UNEMAT) and Federal University of Mato Grosso (UFMT) undertook to carry out the planning, training, and supervision. The same institutions were also responsible for preparing and curating the database. The implementation was by the primary health care system at the municipality level. All procedures were monitored by the Principal Investigator (PI) of the LPEP program and NF representatives. At the beginning of the study, the project also received technical support by the MoH [13].

A total of 16 municipalities were included in PEP-Hans—7 from the state of Mato Grosso, 2 from the Tocantins state, and 7 from the state of Pernambuco. Together, the study sites counted approximately 1 million inhabitants, had 800 new cases per year, and were served by 200 primary health care facilities with at least 200 medical doctors, 200 registered nurses, and 2000 community health workers (CHW). All of them were classified as priority municipalities for leprosy by the MoH. The intervention was based mainly on visits to the home of newly diagnosed patients to enroll household, neighbor, and social contacts [13]. At the end of LPEP program 17 217 contacts of 2669 index cases received SDR in Brazil [12].

The leprosy risk status is related to several aspects, and two of them are: (1) leprosy risk is increased in areas where there are previous records of the disease [14, 15] and (2) the social context [16]. Therefore, it is essential to carry out analyses integrating contextual information with geographic location, which is facilitated by using spatial analysis techniques of geographic data [17, 18]. This approach helps decisively to achieve an effective design of intervention strategies in the medium and long term.

The objective of the study reported here was to analyze changes in the spatial and temporal distribution of leprosy index cases (IC), co-prevalent cases among contacts of leprosy patients (CP), and the factors associated with the occurrence of the disease during the implementation of the LPEP program in Alta Floresta-MT, Brazil.

## Methods

### Study design and area

This study was carried out in the city of Alta Floresta. The city is the most endemic among the 16 municipalities participating in the PEP-Hans program in Brazil [13]. Founded in 1979 in the Brazilian Amazon basin, Alta Floresta had an urban area of 29.5 km^2^ and an estimated population of 51 615 inhabitants in 2018, of which 87% were living in the urban area across 69 census tracts. Precarious housing conditions, a low proportion of households with garbage collection and connected to the sewerage system are indicative of an urban area with infrastructure deficiencies [19], indicating favorable conditions for the spread of infectious diseases. For the spatial analysis, 2 census tracts were excluded because they were characterized by small farms close to the urban area, and 5 others were adjusted to consider the current inhabited urban perimeter.

**Fig. 1:**
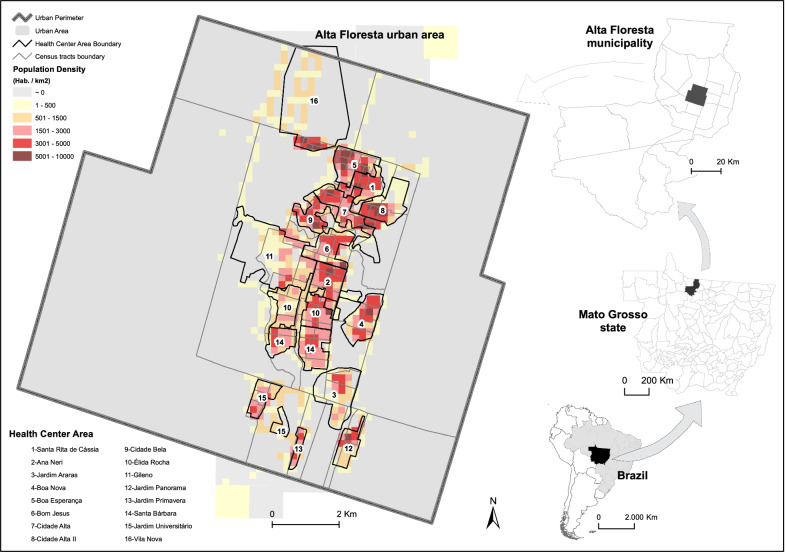
Census tracts, health facility catchment areas and population/10,000 m^2^, urban area of the municipality of Alta Floresta, Mato Grosso, Brazil. Source: Adapted from IBGE, 2010

**Fig. 2:**
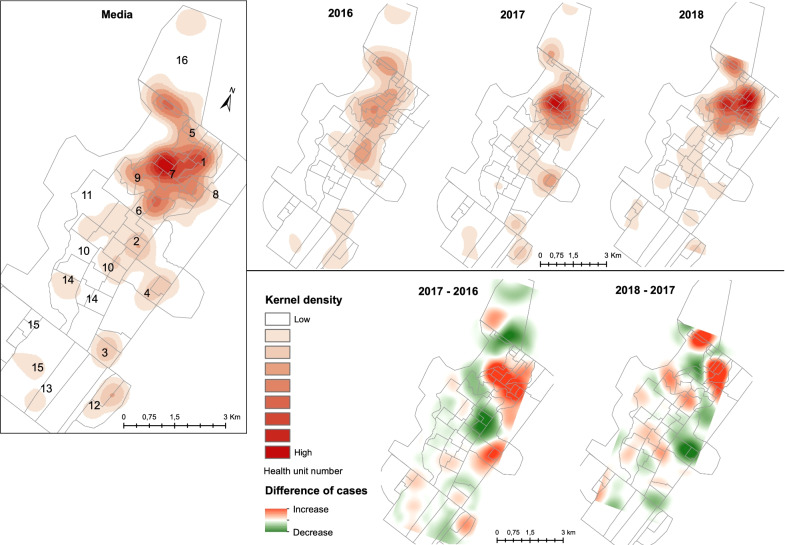
Density and mean number of leprosy index cases per square kilometer and difference in annual number of cases, Alta Floresta, Mato Grosso, Brazil, 2016–2018

The leprosy program in Brazil is decentralized and in Alta Floresta, 16 primary health care centers are located in the urban area. The primary care center number “2” is the reference center for leprosy diagnosis and assistance for patients with complications during treatment. It is the only center with a dermatologist specialized in leprosy. Each primary health center is staffed with a general practitioner, nurse, technical nurses, administrative staff, and around 8 CHW (Figs. 1, 2).

### Data sources

The LPEP program established a database of IC and their contacts. These could be linked to the new cases registered in the Sinan of the state of Mato Grosso. The geographic coordinates of their houses were identified based on the address of the IC recorded in Sinan. ICs among imprisoned individuals were excluded from the spatial analysis. For the automatic geocoding of ICs, the software *Google Earth* was used after the CHWs at each health center confirmed and completed the address, if necessary. The spatial location of household and neighbor contacts was assumed to be the same as that of the IC; addresses of contacts were not systematically collected. The study excluded social contacts, as this group of people most often met at work or social places, thus generally residing at a certain geographic distance from the index patients.

The database of the Brazilian Institute of Geography and Statistics (IBGE) was used for the identification of socioeconomic variables included in the demographic census (2010) and available by census tract. The data selection included socioeconomic characteristics identified based on previous studies on the risk of contracting leprosy [20–22], such as (1) proportion of households without trees in the surroundings; (2) proportion of households without piped water supply; (3) proportion of households without connection to the sewer or septic tank; (4) proportion of households without garbage collection; (5) proportion of households without paving in the surroundings; (6) proportion of illiterate people aged over 60 years; and (7) income from salary (average income of the household head of each sector divided by the minimum wage in 2010 equivalent to ≈ USD 290.00). The spatial data obtained from the IBGE website were organized in shapefiles, by census tracts.

### Data analysis

An epidemiological profile was established for all ICs diagnosed in the study period. For the spatial analyses, only data from urban areas were included due to difficulties in georeferencing the residences of the ICs in rural areas. The NCDR/10 000 inhabitants in general and among those under 15 years of age, and the proportions according to the characteristics of the cases, being: sex (male, female), age (< 15 years, 15–59 years, ≥ 60 years), race/color (white, black, yellow, brown, indigenous), schooling (illiterate, 1st to 9th year of elementary school, 1st to 3rd year of high school, higher upper, full superior), operational classification (paucibacillary, multibacillary), clinical form (indeterminate, tuberculoid, borderline, virchowian), detection mode (referral, passive case detection, collective examination, contact examination, others), bacilloscopy (positive, negative, unrealized, ignored), affected nerves (0 nerves, 01–04 nerves, 05–09 nerves, ≥ 10 nerves), number of skin lesions (0 lesions, 01–04 lesions, 05–09 lesions, ≥ 10 lesions), and degree of disability (0, Grade 1, Grade 2). The proportions of contacts by sex (male, female), type (household, neighbor), contacts that received SDR, contacts that received BCG and the number of doses/scars were calculated [4]. We considered as CP all those patients diagnosed among contacts in the frame of the screening prior to SDR-PEP administration.

For the spatial analysis, the NCDR was calculated by census tract. The estimates of the population per census tracts were obtained from the census data and updated based on the population growth estimates provided by the health center (data available from the health department of the municipality). In addition to the NCDR, the Empirical Bayesian Local Smoothing model was applied based on a neighborhood matrix defined in 1418 m [23], using the software GeoDa 1.12. This distance is equal to the radius of a circle with an area equal to the average of 25 census tracts (6 313 673 m^2^) or 2 neighbors on each side of each census tract. In the point analysis, the case-points obtained by georeferencing were taken into account. A thematic map was drawn with proportional symbols corresponding to the number of household contacts and neighbors, as well as maps that indicated the origin of CP cases.

Kernel analysis considered the IC locations obtained by georeferencing the place of residence to detect and map “hot areas” or clusters of cases, as well as to identify areas of increasing or decreasing number of cases. A bandwidth of 1000 m, a spatial resolution of 50 m, and an adaptive radius were used. The relationship of the CP cases with their respective IC was presented on a point map using vectors.

The SCAN spatial scanning technique was used, with SatScan software version 9.6 [24] to verify changes in the distribution of cases over time and in cluster formation. Through the Discrete Poisson model and retrospective analysis, with application of points with planar coordinates (X, Y) of the centroids of each census tracts and Monte Carlo simulation, the statistical significance of the identified clusters was tested. For the construction of all maps ArcGis 9.2 (ESRI, Redlands, CA, USA) was used.

Factor analysis was performed for the selection of socioeconomic indicators to be included in the model. It was based on principal component analysis and VARIMAX-type data rotation. This statistical technique of multivariate data analysis defines the representative factors of the original variables through the correlation/similarity between them, and so we were able to reduce the number of variables (7 variables to 2 factors) *input* to the model [25]. To adjust the model, Bartlett’s test of sphericity and the Kaiser–Meyer–Olkin (KMO) measure of sample adequacy were used [26].

Spatial regression was applied to analyze associations with NCDR at census tract level using the software GeoDa 1.12.159. The mean NCDR of the 67 census tracts was used as the dependent variable, and the independent variables were the scores of the rotated factors. The choice of the best model was guided by the “Spatial Regression Decision Process” [27]. All statistics considered a significance level of 5%. In the analysis, tests were applied for non-collinearity among the independent variables by means of the conditional number test, presence of heteroscedasticity by the Breush-Pagan, Koenker-Bassett and White’s Robust tests, and test for normality of the residual errors by the Jarque–Bera test [27]. Spatial autocorrelation was identified by means of Lagrange Multipliers (ML).

## Results

### Epidemiological profile

From 2016 to 2018, 304 new leprosy cases were identified in the municipality of Alta Floresta, including 266 (87.5%) in the urban area, of which 258 (96.9%) could be georeferenced.

The mean NCDR was 20.01 cases/10 000 inhabitants, and in people under 15 years old, it was 11.8 cases/10 000 inhabitants. Among the 304 IC, 159 (52.3%) were male, 217 (71.3%) were between 15 and 59 years old, 168 (55.2%) were of mixed race, and 180 of the 273 adults (≥ 18 years, 65.9%) had 0 to 9th-grade schooling. The Additional file 1 contains maps displaying the annual detection rates calculated by the Local Empirical Bayesian method. In 2016, 2 of the 67 urban census tracts had up to 4 cases/10 000 inhabitants, while the corresponding value was 4 in 2017 and 6 in 2018. All other tracts reported more than 4 cases/10 000 inhabitants.

Overall, 265 (87.1%) patients were classified as multibacillary (MB), 202 (74.2%) had a borderline clinical form, 152 (52.6%) were diagnosed by passive case detection, for 218 (71.7%) bacilloscopy was not performed, 273 (78.6%) had 0 to 4 nerves affected, and 228 (75%) had 0 to 4 skin lesions at the time of diagnosis. Among the 181 (59.5%) patients evaluated for physical disabilities, 15 (8.4%) were rated as grade 2.

During the LPEP program, 2167 contacts (duplicate cases were excluded) were listed, and 2076 (95.8%) were examined for signs of leprosy. Among the contacts, 1597 (76.9%) were adults aged 15–59 years, and 1109 (53.4%) were female. Regarding the total number of contacts examined, 1252 were household or neighborhood contacts. In the frame of the contact screening, 55 new (CP) cases were diagnosed, of which 52 (94.5%) were neighbors. Among the examined contacts, 1598 (76.9%) were given SDR, which translates to an average of 8.9 contacts per IC. A total of 363 (17.4%) contacts received a BCG vaccination. Among the contacts, 243 (12.3%) did not have any vaccination scars, and of them, 34 (14%) received the first dose; 1210 (63.7%) had 1 scar, and 329 (26.1%) received the second dose, and 474 (24%) already had 2 scars indicating a full previous BCG vaccination.

### Spatial analysis

**Fig. 3:**
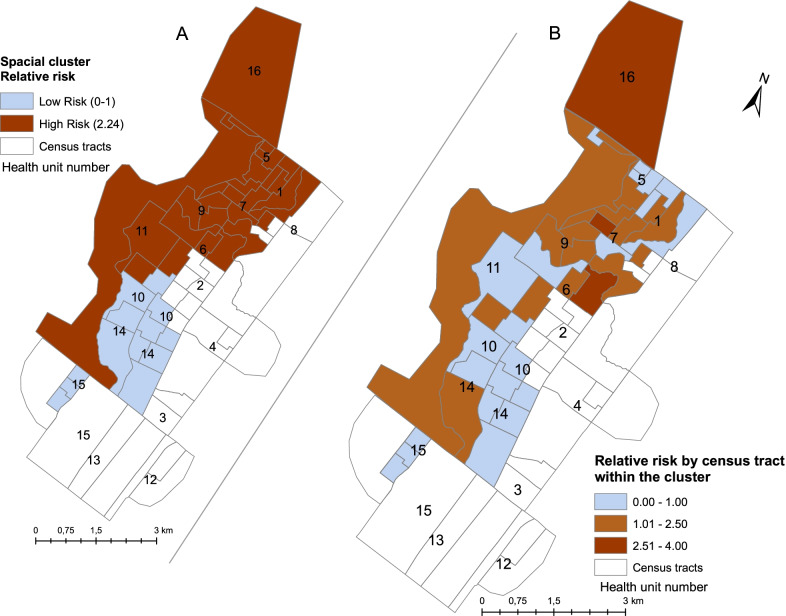
Spatial clusters and relative risk of leprosy detection in Alta Floresta, Mato Grosso, 2016–2018

A purely spatial scan analysis identified 2 statistically significant IC clusters (Fig. 3A). The low-risk cluster is formed by 13 sectors in the south of the urban area [relative risk (*RR*) = 0.21; population 10 502, *P*-value < 0.001], corresponding to 3 health center (1, 10, and 14). The large high-risk cluster (*RR* = 2.24; population 27 427, *P*-value < 0.001) covering the northeastern area of the city included 29 census tracts, corresponding to seven health centers (1, 5, 6, 7, 8, 9, 11, and 16). Simultaneously, the RR for leprosy detection was also calculated for each census tract within the clusters (Fig. 3B). Overall, 23 sectors presented a *RR* < 1.0, in which the *RR* was below the average detection rate. 27 sectors had a *RR* between 1.0 and 2.5, close to the average detection rate, and 6 sectors had a *RR* between 2.5 and 3.42 times above the average detection rate. An estimated 33 600 people lived in the high RR areas. The scan analysis estimate that 1.8 more cases were found than expected, considering the incidence in the urban area.

Changes in the spatial pattern of the disease in the study period can be observed in Kernel maps (Fig. 2), that shows the areas with an elevated IC density over the 3 years of the study. There are highest density of cases in the northeast region of the urban area that corresponds to the catchment areas of health centers 1, 6, 7, and 16. The dispersion at the beginning (2016) extending to the central region of the city, and evidence of a concentration of cases in the northern region in the last year. There was an increase in the density of cases in 2017 compared to 2016 mainly in the northeast area (center 7, 1, and part of 8), in addition to the center-east area (center 4), and a reduction in north and central areas (center 16 and 2 respectively). When comparing the year 2018 to the year 2017, there was an increase again in the northeast area (but concentrated in center 8), as well as in the north area (center 5 and 16). There was also a reduction in the center-east area (center 4), in addition to many small dispersed areas.

**Fig. 4:**
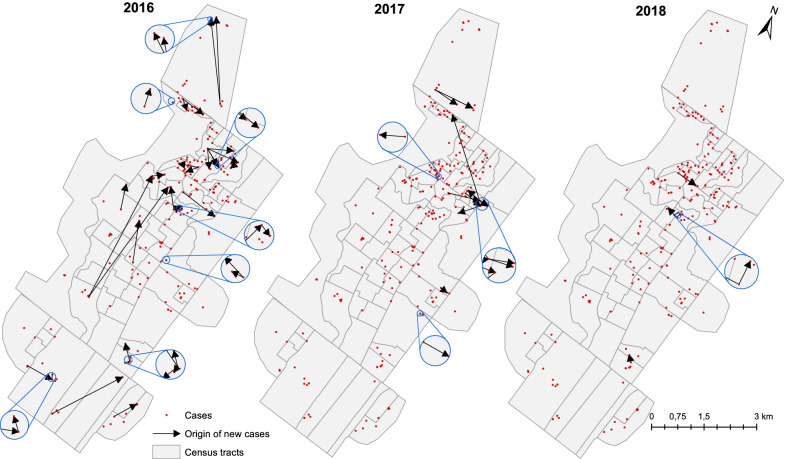
Georeferenced leprosy index cases and vectors linking co-prevalent (leprosy positive) contacts. Alta Floresta, Mato Grosso, 2016–2018

Figure 4 shows the distribution of ICs by year, with arrows indicating the CP cases linked to them. It can be observed that up to five new leprosy cases were linked to a single IC during the intervention period. There were 35 CPs identified in 2016, 13 in 2017, and 7 in 2018. Note that 1 CP case in the year 2016, 1 in 2017, and 3 in 2018 do not have origin markings in the figure because they were related to some of the 11 ICs diagnosed in the public jail, hence excluded from the spatial analyses.

The average number of household and neighbor contacts per IC was 7. Over the three years of the study, there was a reduction in the average number of screened contacts/IC, as well as a change in the spatial pattern of contacts examined per IC, similar to the change in the spatial pattern of the disease. In 2016, 16 IC had no contacts evaluated, and in the years 2017 and 2018, there were 22 and 33 cases, respectively (Additional file 2).

In the factor analysis, 2 factors were identified that together explained 74.5% of the variation in the data. Bartlett’s test of sphericity was equal to 160.758 with significant correlations (*P* < 0.001), and the KMO with the value 0.683 indicated the adequacy of the factor analysis method. The variable ‘Proportion of households without trees in the surroundings” was excluded because it did not explain the variation of the data between the census tracts, thus leaving 6 variables grouped into 2 factors. Factor 1 was called “poverty” and included the variables: proportion of households without a link to the sewage system or septic tank, proportion of households without paving in the surroundings, proportion of non-literate people over 60 years old, and monthly income. Factor 2 was named “water and garbage”, which included the following variables: the number of households without a link to the centralized water distribution system and the number of houses without garbage collection (Table 1).

**Table 1:** Correlation between variables and VARIMAX rotated factors extracted by Principal Component Analysis by census tract, Alta Floresta, Mato Grosso, according to the 2010 census

Variables	Factor	
	1 (poverty)	2 (water and garbage)
Proportion of households without a link to the centralized water distribution system	0.125	**0.903**
Proportion of households without sewerage system or septic tank	**0.794**	0.122
Proportion of households without garbage collection	0.020	**0.897**
Proportion of households without paving in the surroundings	**0.791**	-0.413
Proportion of illiterate people over 60 years old	**0.797**	0.167
Monthly income	**-0.849**	-0.100

The spatial regression with the average leprosy detection rate as the dependent variable showed significance of the variable “poverty” and non-significance of the variable “water and garbage” in the modeling process. The regression diagnosis was positive for non-multicollinearity and normality of residuals but not positive for heteroscedasticity. The diagnosis of spatial dependence points to Classical Regression as the most suitable model since it did not show significance for ML (Table 2).

**Table 2:** Classical regression model, considering as dependent variable the average detection rate by census tract

Variable	Test	GL	Coefficient	Probability
Classical regression				
Constant			13.0747	0.0000
Poverty			3.3368	0.0109
Water and garbage			2.0661	0.1069
Regression diagnostics				
Multicollinearity	Conditional number		1.0152	
Normality of residuals	Jarque–Bera	2	5.9743	0.0504
Heteroscedasticity	Breuch-Pagan	2	0.1901	0.9093
	Koenter-Basset	2	0.1633	0.9216
	White’s Robust	5	6.2055	0.2867
Spatial dependence diagnostics				
	ML (lag)	1	0.0679	0.7945
	ML robust (lag)	1	0.0005	0.9822
	ML (error)	1	0.0708	0.7902
	ML robust (error)	1	0.0034	0.9535

GL: Degrees of freedom, ML (lag): Lagrange Multipliers (Spatial Lag Model), ML robust (lag): Lagrange Multipliers robust (Spatial Lag Model), ML (error): Lagrange Multipliers (Spatial Error Model), ML robust (error): Lagrange Multipliers robust (Spatial Error Model)

## Discussion

In this study, we highlight the impact of the LPEP program on the spatial pattern of leprosy during the three years of the intervention. The analysis of the spatial and temporal distribution of new leprosy cases in Alta Floresta highlights a heterogeneous distribution, as well as changes in the spatial pattern over the study period. In the last year, new cases were even more concentrated in the northeast of the urban area compared to previous years. The areas identified as high risk also corresponded to those with a higher NCDR, above-average number of screened contacts, and CP cases. It is important to highlight that SDR-PEP cannot be dissociated from active case finding among contacts. The reduction of the risk areas and progressive concentration of cases facilitate the identification of geographic focus areas for transmission reduction efforts.

The Kernel method identified a change in the density pattern of cases over the study period, but the spatio-temporal evolution of the NCDR did not show statistical significance. The density of cases refers to the concentration of an absolute number of cases, and the risk areas were estimated as rates. Risk areas did not change over time, possibly because of the short period of follow-up, in addition to the small number of census tracts. It is conceivable that with the continuation of the intervention, the local leprosy distribution would become even more focal and thus spatially restricted. This finding partially differs from other studies, which associate a high density of leprosy cases with population density [28, 29].

The areas identified as having an elevated risk and the change in the density pattern of cases may result from the intensification of contact tracing and screening activities by health workers, considering that the LPEP intervention was carried out in the period concomitant to the study. Changes in the spatial pattern are expected after case and contact surveillance [8, 30, 31].

Low-risk areas, identified by purely spatial scan, overlap with a homogeneous area in the city presenting the low values of the factor ‘poverty’ (Additional file 3). On the other hand, areas presenting good socioeconomic conditions represent protection from leprosy [32]. This corroborates findings from other studies that indicate a relationship between socioeconomic and demographic indicators and the risk of leprosy [15, 33]. Priority areas for intensified leprosy control activities can thus be identified with this method.

The proportion of contacts/IC declined over the analysis period, which can be explained by the repeated targeting of the same contacts over the course of the intervention. Repeated screenings were excluded from analysis (Additional file 2). There also was a decrease in CP cases among the examined contacts, even in the short period of the study. Our study suggests that active surveillance of contacts can change the disease pattern and contribute to disease risk reduction, with detection of missed cases at the beginning and a gradual reduction in the NCDR over time, reflecting the impact of the preventive intervention. This complements other LPEP results modeling showing the effects of the intervention combining surveillance with SDR in the long-term [4, 5].

The proportion of CP cases was higher among neighborhood contacts compared to household contacts, showing the relevance of contact tracing outside the patient’s home. It is known that household contacts and neighbors represent the main risk groups for new leprosy cases [34, 35]. Many studies emphasize the transmission risk in the home environment and show a greater risk of illness among people who live directly with the patient [36–39]. In this sense, the distance between households and family structures likely explains the differences between the areas in terms of leprosy epidemiology. Therefore, surveillance strategies must consider these aspects [34, 35]. In modeling studies on the long-term impact of the LPEP program, it has been shown that contact tracing, screening, and provision of SDR-PEP potentially reduce the number of new cases, with impacts increasing over time [5, 12].

In this study, factor and multiple regression analysis showed that a neighborhood matrix of the census tracts did not statistically significantly influence the average detection rates. However, the conditions related to “poverty” have similar patterns to the distribution of the disease and were related to the occurrence of leprosy in the urban area of Alta Floresta, showing a higher risk of disease in areas with higher levels of poverty. While there are multiple possible factors involved, studies have shown that the risk of leprosy increased considerably in the outskirts of cities. Typically, Brazilian city outskirts have poor socioeconomic and demographic indicators, a high population density, and little access to municipal services such as sewage and piped water [40–43]. Several studies identified variables related to poverty as a determining factor for the transmission and occurrence of leprosy. The studies also suggest that actions focused on improving these conditions can contribute to the reduction of disease incidence, in addition to the other actions already recommended by the MoH and WHO [44–46].

Local economic and managerial conditions have an important influence on the availability and quality of health services, acting as a determinant of the likelihood of case diagnosis. The heterogeneous distribution of both disease and poverty suggests that increased social deprivation is associated with a higher risk of developing leprosy and maintaining the chain of transmission of *Mycobacterium leprae* [15, 33, 42, 47, 48]. On the other hand, some studies suggest that screening activities are intensified in areas with more vulnerable populations, contributing to an apparent increase in the rate of leprosy [49, 50].

The predominance of MB cases and important rate of child patients indicates intense transmission in the study area, while a high proportion of physical disabilities suggests late diagnosis [4]. The predominance of cases among individuals classified as brown race is possibly linked to the association of poverty with this race in the state of Mato Grosso. The average monthly household income of people of brown race/color corresponds to 63.1% of the income of white people. Although the state shows improvements in all dimensions of the Municipal Human Development Index (MHDI) and also reduction of poverty and vulnerability indicators over time, progress is unequal between municipalities [51, 52].

The limitations of this study are related to possible underreporting and eventual errors due to database completeness and consistency problems. The use of cartographic bases from different sources to generates a spatial displacement can cause errors in the allocation of records in the respective census sectors, compromising the effectiveness of the analysis. We tried to solve this problem with site visits to verify the location of the geocoded records, and we found no errors.

The difference in the temporality of census information also constitutes a limitation, with the need for updated data on the characteristics of the Brazilian population. On the other hand, the approach by area of coverage of the primary care center to estimate the population of the census sectors was an innovation and proved to be effective for calculating detection rates. Another strong point was the use of disaggregated data at the census sector level and a high percentage of georeferencing, reflecting the effectiveness of the strategies adopted by the health team to improve address information when searching for contacts.

This study provides evidence about the focal occurrence of leprosy and its spatial heterogeneity. The LPEP activities appear to have influenced the spatial dynamics of leprosy and results underline the relevance of systematic contact surveillance for leprosy elimination. The change in the spatio-temporal pattern of leprosy cases even in a relatively short period indicates that systematic contact surveillance can contribute to leprosy control and influence the local epidemiology.

The spatial analyses revealed priority areas for interventions, and highlighted poverty as a risk factor for leprosy at census tract level in the urban area of Alta Floresta. Hence, we conclude that poverty is an important factor to identify critical areas for leprosy surveillance. The sustained hyperendemicity in the study site also suggests that innovative strategies should be encouraged to achieve greater effectiveness of leprosy control interventions.

For the successful interruption of *M. leprae* transmission and elimination of leprosy it is necessary to use available and recommended interventions. The WHO recommends SDR-PEP, and some national programs have already included or are considering to include such interventions in the routines of their health services. It is also important to create partnerships with other institutions in order to develop new and more effective tools and initiatives for the prevention and control of leprosy [12, 53, 54].

## Conclusions

This study shows the relevance of systematic contact tracing, screening and single-dose rifampicin administration to contacts of leprosy patients for leprosy surveillance, as well as its impact on the distribution of new cases in an urban environment. We were able to reconstruct the changing spatial risk of the diseases over the duration of the intervention. The disease distribution was partly explained by poverty indicators. LPEP influenced the spatial dynamic of the disease and results highlighted the relevance of systematic contact surveillance for leprosy elimination.

## Supplementary Information

**Additional file 1.** Annual detection rate by the Local Empirical Bayesian method, Alta Floresta, Mato Grosso, 2016–2018.

**Additional file 2. **Household contacts and neighbors examined per index case per year, Alta Floresta, Mato Grosso, 2016–2018.

**Additional file 3. **Spatial distribution of the factors extracted from the analysis, Alta Floresta, Mato Grosso.

## Data Availability

The datasets used and/or analyzed during the current study are available from the corresponding author upon reasonable request.

## Abbreviations

NTD: Neglected tropical disease
NCDR: New case detection rate
LPEP: Leprosy post-exposure prophylaxis
SDR-PEP: Leprosy post-exposure prophylaxis with single dose rifampicin
BCG: Bacille Calmette-Guérin
MoH: Ministry of Health
NF: Novartis Foundation
UNEMAT: State University of Mato Grosso
UFMT: Federal University of Mato Grosso
CHW: Community health workers
CONITEC: National Commission for Technology Incorporation
WHO: World Health Organization
IC: Index case
CP: Co-prevalent case
Sinan: Notifiable diseases information system
IBGE: Brazilian Instituto of Geography and Statistics
KMO: Kaiser–Meyer–Olkin
ML: Lagrange multipliers
MB: Multibacillary
MHDI: Municipal Human Development Index
*RR*: Relative risk
PI: Principal investigator
