# Supplementary figures and images for: Spatio-temporal analysis of leprosy risks in a municipality in the state of Mato Grosso-Brazilian Amazon: results from the leprosy post-exposure prophylaxis program in Brazil
Source: Infect Dis Poverty. 2022 Feb 22;11:21. doi: 10.1186/s40249-022-00943-7 (PMC8862266; doi:10.1186/s40249-022-00943-7)

2016

2017

2018

**Detection rate  
/10,000**

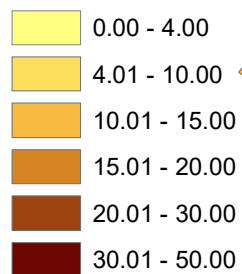

0 0,75 1,5 3 Km

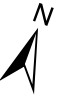

Supplement: Supplementary file 1 — Additional file 1. Annual detection rate by the Local Empirical Bayesian method, Alta Floresta, Mato Grosso, 2016–2018. [file 40249_2022_943_MOESM1_ESM.pdf]

2016

2017

2018

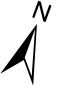

**Examined contacts**

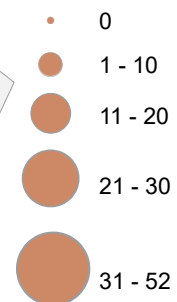

Health unit area

Census tracts

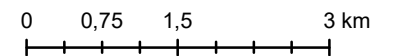

Supplement: Supplementary file 2 — Additional file 2. Household contacts and neighbors examined per index case per year, Alta Floresta, Mato Grosso, 2016–2018. [file 40249_2022_943_MOESM2_ESM.pdf]
