# Supplementary material for: Spatio-temporal analysis of leprosy risks in a municipality in the state of Mato Grosso-Brazilian Amazon: results from the leprosy post-exposure prophylaxis program in Brazil
Source: Infect Dis Poverty. 2022 Feb 22;11:21. doi: 10.1186/s40249-022-00943-7 (PMC8862266; doi:10.1186/s40249-022-00943-7)
Supplement: Supplementary file 3 — Additional file 3. Spatial distribution of the factors extracted from the analysis, Alta Floresta, Mato Grosso. [file 40249_2022_943_MOESM3_ESM.pdf]

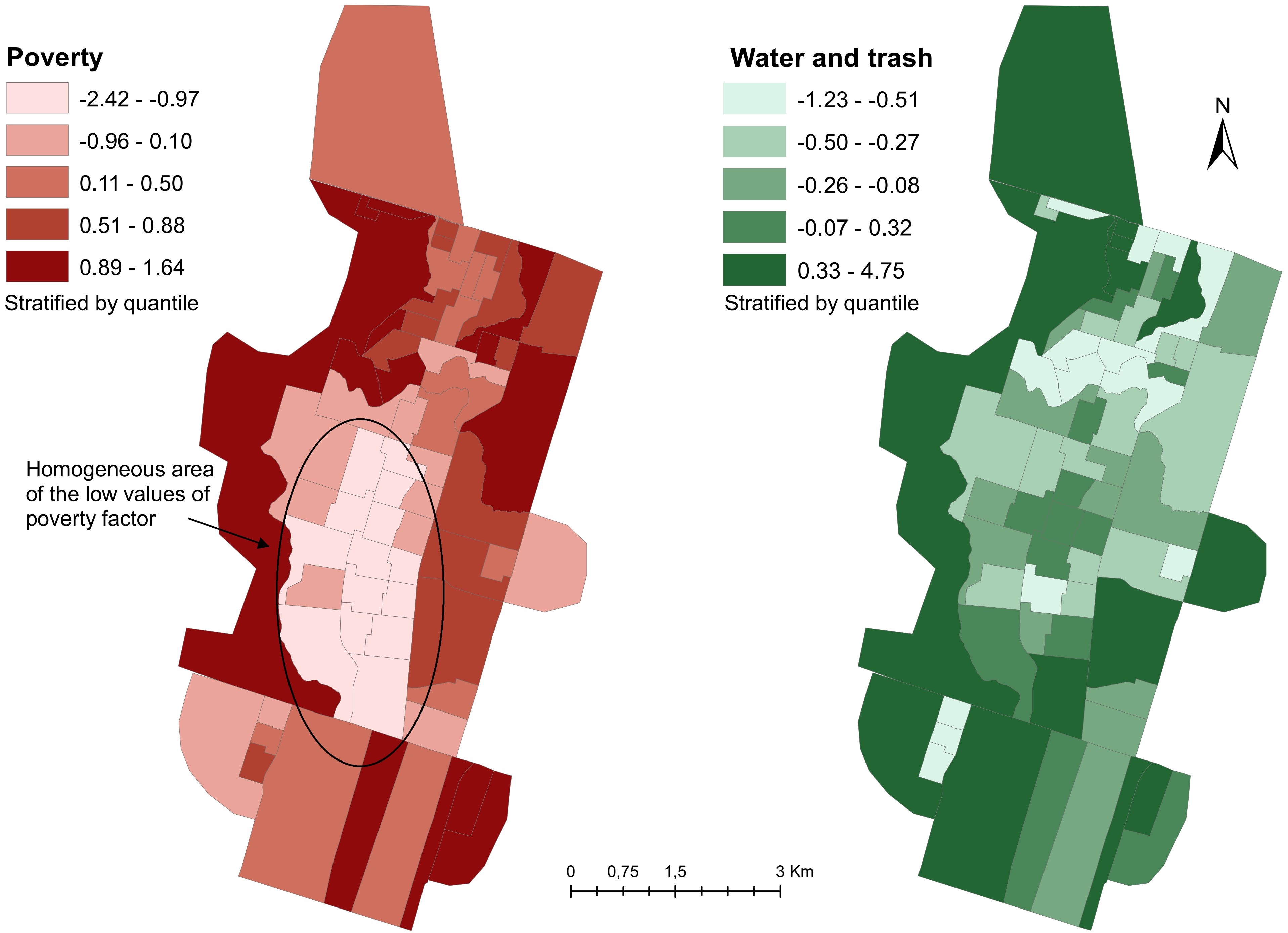

Poverty
-2.42 - -0.97
-0.96 - 0.10
0.11 - 0.50
0.51 - 0.88
0.89 - 1.64
Stratified by quantile
Homogeneous area of the low values of poverty factor
Water and trash
-1.23 - -0.51
-0.50 - -0.27
-0.26 - -0.08
-0.07 - 0.32
0.33 - 4.75
Stratified by quantile
N
0
0,75
1,5
3 Km
